# Supplementary material for: A Likelihood Approach for Real-Time Calibration of Stochastic Compartmental Epidemic Models
Source: PLoS Comput Biol. 2017 Jan 17;13(1):e1005257. doi: 10.1371/journal.pcbi.1005257 (PMC5240920; doi:10.1371/journal.pcbi.1005257)
Supplement: S1 File — (TAR.GZ) [file pcbi.1005257.s014.tar.gz › HSPH_Online-SI-Revision/IPoi09/n100-mild/n100-mild_BENCH_table0.pdf]

1	R0	{1.24125, 3.38982}	{1.67405, 7.28757}	{{1.67405, 7.28757}, {1.67405, 7.28757}}	{34.8681, 114.984}	inf	1822.	7484.21	{5042., 9285.}	310.769	0.601138	newly	3621.	6739.89	{4077., 8701.}	87.6306	0.261354	Reff	0.993708	0.877899	{0.66967, 1.26555}	22.2538	ar	34 926.	67 952.9	{67 276., 68 619.}	94.5624
2	R0	{1.29554, 4.51878}	{2.0072, 10.6431}	{{2.0072, 10.6431}, {2.0072, 10.6431}}	{54.9315, 135.53}	inf	2837.	12 803.8	{9131., 15 397.}	352.892	0.645798	newly	4247.	7855.15	{5268., 9910.}	87.8492	0.268447	Reff	0.958635	0.826644	{0.584617, 1.14637}	24.4201	ar	42 828.	79 852.6	{79 507., 80 196.}	86.4496
3	R0	{1.2443, 3.64292}	{1.93419, 8.56054}	{{1.93419, 8.56054}, {1.93419, 8.56054}}	{55.444, 134.991}	inf	2191.	12 282.	{9089., 14 125.}	460.566	0.742887	newly	4114.	9307.32	{6333., 11 305.}	126.235	0.344815	Reff	0.95617	0.84062	{0.628921, 1.07891}	19.7593	ar	37 087.	77 863.7	{77 421., 78 275.}	109.949
4	R0	{1.38465, 3.43331}	{2.21076, 7.20429}	{{2.21076, 7.20429}, {2.21076, 7.20429}}	{59.662, 109.835}	inf	3745.	14 027.4	{8546., 18 443.}	274.563	0.558204	newly	6729.	11 899.	{6416., 16 925.}	79.8877	0.238684	Reff	0.908206	0.734454	{0.460744, 1.1423}	33.3297	ar	49 511.	84 592.2	{84 325., 84 880.}	70.8553
5	R0	{1.25409, 4.1068}	{1.83586, 7.22097}	{{1.83586, 7.22097}, {1.83586, 7.22097}}	{46.3898, 75.8296}	inf	2471.	9361.8	{5786., 12 215.}	278.867	0.561426	newly	3936.	8085.18	{4553., 11 223.}	107.379	0.300807	Reff	0.961511	0.767119	{0.573578, 0.977045}	24.7703	ar	38 571.	74 596.6	{74 099., 75 039.}	93.4007
6	R0	{1.28821, 4.2016}	{1.93462, 9.38215}	{{1.93462, 9.38215}, {1.93462, 9.38215}}	{50.1789, 123.299}	inf	2769.	11 882.6	{9661., 14 150.}	329.128	0.626441	newly	4394.	8194.42	{6270., 10 300.}	86.7109	0.261679	Reff	0.936864	0.807931	{0.627242, 1.02428}	20.5553	ar	41 468.	77 825.3	{77 388., 78 158.}	87.6756
7	R0	{1.24369, 2.56007}	{2.12684, 8.23415}	{{2.12684, 8.23415}, {2.12684, 8.23415}}	{71.0105, 221.638}	inf	2411.	14 775.8	{10 897., 17 403.}	512.848	0.773146	newly	6137.	12 080.8	{8983., 14 404.}	98.0456	0.290701	Reff	0.952269	1.00757	{0.64288, 1.56491}	29.5205	ar	38 603.	82 827.	{82 496., 83 119.}	114.561
8	R0	{1.232, 3.53942}	{1.93568, 10.0274}	{{1.93816, 10.0503}, {1.93816, 10.0503}}	{57.1165, 183.307}	inf	1816.	11 310.8	{7890., 14 139.}	523.831	0.778639	newly	3274.	7381.84	{4749., 9695.}	128.097	0.349513	Reff	0.934361	0.838906	{0.593038, 1.17701}	25.3408	ar	34 555.	77 857.9	{77 454., 78 370.}	125.316
9	R0	{1.36262, 3.85631}	{2.33832, 9.79994}	{{2.33832, 9.79994}, {2.33832, 9.79994}}	{71.6047, 154.127}	inf	3694.	16 921.5	{12 674., 20 677.}	361.715	0.657785	newly	6191.	11 399.7	{7521., 14 437.}	89.1523	0.279608	Reff	0.926772	0.936615	{0.550838, 1.6842}	37.2281	ar	48 410.	86 902.8	{86 650., 87 184.}	79.5141
10	R0	{1.28702, 4.67968}	{1.87927, 9.84456}	{{1.87927, 9.84456}, {1.87927, 9.84456}}	{46.0172, 110.368}	inf	2625.	8613.49	{5568., 12 839.}	228.133	0.490226	newly	3765.	5592.51	{3385., 8638.}	56.0382	0.179226	Reff	0.941571	0.721365	{0.546041, 1.03435}	30.6966	ar	41 092.	75 982.9	{75 584., 76 490.}	84.9091
11	R0	{1.35752, 3.47129}	{2.62879, 15.1183}	{{2.62879, 15.1183}, {2.62879, 15.1183}}	{93.6465, 335.524}	inf	3108.	20 173.	{7468., 25 134.}	551.969	0.768719	newly	6127.	9740.02	{4557., 11 600.}	73.2221	0.261283	Reff	1.02022	1.43875	{0.911454, 2.29507}	46.1835	ar	44 669.	90 813.7	{90 653., 90 996.}	103.304
12	R0	{1.35929, 5.83647}	{1.98131, 11.1772}	{{1.98131, 11.1772}, {1.98131, 11.1772}}	{45.7607, 91.5062}	inf	3507.	12 171.3	{7567., 14 400.}	247.057	0.53004	newly	4123.	7379.21	{4922., 9216.}	79.8706	0.246148	Reff	0.975236	0.93451	{0.62974, 1.43992}	29.1212	ar	46 715.	79 152.9	{78 853., 79 488.}	69.4379
13	R0	{1.28019, 3.29908}	{2.06386, 8.81239}	{{2.06386, 8.81239}, {2.06386, 8.81239}}	{61.2151, 167.117}	inf	2522.	14 026.	{11 507., 16 251.}	456.148	0.738447	newly	5146.	10 439.2	{7771., 12 829.}	103.427	0.298706	Reff	0.960053	0.86818	{0.599469, 1.18218}	24.801	ar	40 941.	81 347.9	{81 053., 81 619.}	98.6955
14	R0	{1.36661, 3.48318}	{2.26582, 8.12562}	{{2.26582, 8.12562}, {2.26582, 8.12562}}	{65.7986, 133.282}	inf	3348.	14 656.6	{8237., 19 634.}	339.532	0.625178	newly	5669.	11 223.5	{5568., 16 490.}	101.176	0.285164	Reff	0.881791	0.762072	{0.446933, 1.23073}	35.1938	ar	48 672.	85 637.7	{85 350., 86 000.}	75.9487
15	R0	{1.35865, 3.84123}	{2.95883, 17.6422}	{{2.93371, 17.1903}, {2.96398, 17.7348}}	{117.778, 359.286}	inf	3969.	23 206.6	{9349., 29 283.}	484.926	0.727906	newly	6860.	9632.96	{4881., 11 563.}	52.9081	0.197289	Reff	0.953229	1.4975	{0.718617, 2.52955}	67.3928	ar	48 598.	93 741.3	{93 556., 93 942.}	92.8912
16	R0	{1.22879, 3.98329}	{1.70777, 8.49961}	{{1.70777, 8.49961}, {1.70777, 8.49961}}	{38.9798, 113.382}	inf	1615.	8286.66	{6661., 9953.}	413.106	0.702559	newly	2786.	6425.88	{4854., 8120.}	130.649	0.353019	Reff	0.98678	0.871461	{0.692706, 1.13726}	21.2736	ar	34 405.	69 566.9	{69 033., 69 995.}	102.2
17	R0	{1.34925, 3.64524}	{2.11099, 8.63718}	{{2.10405, 8.58066}, {2.10405, 8.58066}}	{56.4564, 136.944}	inf	3518.	14 061.	{9311., 16 942.}	299.687	0.590121	newly	6327.	11 053.3	{8294., 13 408.}	75.1652	0.235755	Reff	0.979043	1.00381	{0.595404, 1.64265}	32.339	ar	46 871.	82 428.3	{82 000., 82 605.}	75.862
18	R0	{1.33054, 2.25275}	{2.17292, 6.16453}	{{2.17292, 6.16453}, {2.17292, 6.16453}}	{63.3111, 173.645}	inf	3041.	15 520.3	{11 906., 18 050.}	410.37	0.701699	newly	8646.	15 572.9	{10 667., 19 734.}	80.1164	0.244379	Reff	0.969272	0.844683	{0.555398, 1.22303}	26.6513	ar	44 336.	83 825.5	{83 511., 84 088.}	89.0688
19	R0	{1.28394, 3.69859}	{1.98131, 11.1772}	{{1.98131, 11.1772}, {1.98131, 11.1772}}	{54.3148, 202.202}	inf	2442.	12 351.3	{7880., 14 951.}	405.785	0.684992	newly	4445.	7723.4	{5364., 9178.}	76.2525	0.239888	Reff	0.980378	1.08397	{0.727339, 1.61794}	28.6187	ar	39 558.	79 152.9	{78 853., 79 488.}	100.093
20	R0	{1.2883, 2.26493}	{2.0078, 5.69114}	{{1.98552, 5.56151}, {2.0598, 5.9936}}	{55.8491, 151.272}	inf	2636.	12 825.1	{8892., 15 649.}	387.543	0.675681	newly	7800.	15 146.6	{11 549., 18 353.}	95.6569	0.286683	Reff	1.01925	1.05169	{0.652895, 1.61325}	29.4245	ar	40 768.	80 078.1	{79 129., 81 444.}	96.4239
21	R0	{1.34098, 2.78621}	{1.81179, 4.06746}	{{1.81179, 4.06746}, {1.81179, 4.06746}}	{35.1094, 45.9854}	inf	3142.	6922.52	{3923., 10 712.}	120.365	0.315068	newly	7245.	9116.2	{4686., 15 890.}	47.036	0.165934	Reff	0.972707	0.668287	{0.554245, 0.843606}	33.7649	ar	44 605.	73 634.8	{73 116., 74 236.}	65.0819
22	R0	{1.33783, 5.95911}	{1.88096, 9.64595}	{{1.88096, 9.64595}, {1.88096, 9.64595}}	{40.5978, 61.869}	inf	3350.	9977.32	{6839., 13 149.}	197.83	0.459023	newly	3826.	6725.48	{4274., 9381.}	77.7533	0.23319	Reff	0.988844	0.786172	{0.575085, 1.05234}	27.1112	ar	45 418.	76 140.8	{75 655., 76 538.}	67.6444
23	R0	{1.33359, 3.3069}	{2.76203, 15.0558}	{{2.76203, 15.0558}, {2.76203, 15.0558}}	{107.112, 355.284}	inf	3385.	21 677.8	{7719., 26 755.}	542.689	0.769088	newly	6752.	10 429.9	{4847., 12 403.}	66.4692	0.239585	Reff	0.971427	1.40276	{0.746632, 2.32334}	54.0335	ar	46 245.	92 152.	{91 982., 92 321.}	99.2691
24	R0	{1.30588, 2.56123}	{2.04866, 5.92879}	{{1.98552, 5.56151}, {2.0598, 5.9936}}	{56.8795, 131.482}	inf	2762.	12 552.2	{7938., 15 805.}	354.46	0.644089	newly	6606.	13 092.5	{7109., 18 473.}	98.2981	0.27697	Reff	0.932189	0.82016	{0.534189, 1.24047}	29.6735	ar	42 383.	80 939.8	{79 533., 81 557.}	90.9724
25	R0	{1.29813, 4.51849}	{2.22641, 14.8348}	{{2.22641, 14.8348}, {2.22641, 14.8348}}	{71.509, 228.313}	inf	3099.	15 444.2	{8543., 19 074.}	403.736	0.700211	newly	4627.	7517.37	{4700., 8925.}	71.0737	0.261113	Reff	0.990123	1.28736	{0.791266, 1.84667}	38.9034	ar	43 371.	84 916.4	{84 644., 85 184.}	95.7908
26	R0	{1.33231, 3.99953}	{2.68803, 17.202}	{{2.68803, 17.202}, {2.68803, 17.202}}	{101.757, 330.101}	inf	3222.	22 057.7	{12 750., 26 037.}	585.85	0.806136	newly	5352.	9050.77	{6372., 10 493.}	76.7995	0.258125	Reff	0.96366	1.24175	{0.684587, 2.09083}	42.7316	ar	44 733.	91 430.8	{91 253., 91 588.}	104.392
27	R0	{1.30555, 4.61445}	{2.47315, 17.575}	{{2.46976, 17.5057}, {2.49092, 17.9391}}	{89.4332, 280.87}	inf	3092.	18 176.3	{8381., 22 994.}	490.811	0.74052	newly	4581.	7433.13	{4169., 9086.}	72.8346	0.254238	Reff	0.983993	1.34715	{0.765576, 2.10577}	46.4761	ar	42 624.	88 933.	{88 648., 89 242.}	108.645
28	R0	{1.26819, 5.34969}	{1.68297, 7.5059}	{{1.68297, 7.5059}, {1.68297, 7.5059}}	{32.7065, 40.3053}	inf	2467.	6748.84	{4157., 9475.}	173.565	0.418213	newly	3182.	5758.	{3268., 8798.}	82.9105	0.238861	Reff	0.987438	0.792008	{0.62216, 1.11372}	25.01	ar	38 946.	68 311.3	{67 696., 68 803.}	75.4001
29	R0	{1.19925, 3.00394}	{1.75445, 7.55154}	{{1.75445, 7.55154}, {1.75445, 7.55154}}	{46.2956, 151.388}	inf	1551.	8707.49	{6540., 10 571.}	461.411	0.738174	newly	3362.	7478.1	{5126., 9702.}	122.446	0.334634	Reff	0.975746	0.858751	{0.663691, 1.19562}	23.2541	ar	31 727.	71 490.9	{70 900., 71 883.}	125.332
30	R0	{1.35749, 3.75483}	{2.96225, 15.9111}	{{2.96947, 15.963}, {2.96947, 15.963}}	{118.215, 323.75}	inf	3853.	25 526.2	{12 662., 29 439.}	565.407	0.80283	newly	6557.	11 118.6	{7339., 12 852.}	77.0545	0.261445	Reff	0.934306	1.17171	{0.597576, 2.23384}	45.0392	ar	48 244.	93 776.7	{93 646., 93 970.}	94.38
31	R0	{1.27131, 4.2161}	{2.0072, 10.6431}	{{2.0072, 10.6431}, {2.0072, 10.6431}}	{57.8844, 152.439}	inf	2579.	13 731.1	{10 336., 15 408.}	435.141	0.725278	newly	4228.	8814.85	{6793., 10 188.}	111.88	0.330831	Reff	0.992664	0.984963	{0.678333, 1.37567}	21.491	ar	38 299.	79 852.6	{79 507., 80 196.}	108.498
32	R0	{1.31827, 6.18119}	{1.88067, 9.67971}	{{1.87927, 9.64595}, {1.88096, 9.84456}}	{42.6622, 56.5995}	inf	3314.	10 152.7	{6809., 13 149.}	209.088	0.481799	newly	3614.	6829.76	{4251., 9275.}	92.9063	0.276761	Reff	0.960834	0.823614	{0.58242, 1.3215}	26.4874	ar	45 262.	76 107.2	{75 607., 76 508.}	68.1481
33	R0	{1.20398, 1.35167}	{2.18544, 6.6879}	{{2.0598, 5.9936}, {2.22088, 6.88373}}	{81.5182, 394.788}	inf	1581.	14 966.1	{8835., 18 831.}	848.349	0.956566	newly	7272.	15 771.9	{11 870., 18 759.}	119.896	0.343813	Reff	0.95218	1.16774	{0.652895, 1.75192}	38.7238	ar	33 329.	83 934.4	{81 266., 85 010.}	151.836
34	R0	{1.32776, 3.146}	{2.59133, 11.8519}	{{2.59133, 11.8519}, {2.59133, 11.8519}}	{95.1655, 276.729}	inf	3143.	20 755.8	{15 330., 24 453.}	565.69	0.811915	newly	6043.	11 809.8	{8466., 13 960.}	102.927	0.324973	Reff	0.90054	1.04704	{0.598908, 1.6223}	41.0936	ar	44 872.	90 403.9	{90 193., 90 599.}	101.471
35	R0	{1.279, 1.11336}	{2.82466, 9.2843}	{{2.82466, 9.2843}, {2.82466, 9.2843}}	{120.849, 733.899}	inf	1968.	24 281.2	{18 246., 27 658.}	1134.02	1.06876	newly	7436.	17 233.9	{11 965., 20 344.}	133.805	0.365472	Reff	0.863977	1.03213	{0.578208, 1.59853}	41.3317	ar	41 033.	92 686.6	{92 521., 92 865.}	125.883
36	R0	{1.20029, 1.94717}	{1.71582, 3.97874}	{{1.71582, 3.97874}, {1.71582, 3.97874}}	{42.9505, 104.334}	inf	1687.	8041.22	{5760., 10 066.}	376.658	0.666412	newly	5772.	12 155.7	{7212., 16 843.}	111.23	0.30326	Reff	0.983182	0.847943	{0.641871, 1.23421}	21.8581	ar	33 234.	69 787.	{69 339., 70 243.}	109.987
37	R0	{1.33216, 3.69685}	{2.60082, 15.3681}	{{2.60082, 15.3681}, {2.60082, 15.3681}}	{95.2333, 315.708}	inf	3070.	20 541.1	{9025., 24 695.}	572.343	0.801683	newly	5464.	9448.7	{5259., 11 187.}	82.9882	0.282966	Reff	0.949151	1.25558	{0.735278, 2.19452}	45.2277	ar	45 235.	90 485.7	{90 296., 90 660.}	100.035
38	R0	{1.25434, 2.04314}	{1.98552, 5.56151}	{{1.98552, 5.56151}, {1.98552, 5.56151}}	{58.292, 172.204}	inf	2420.	12 040.5	{4798., 14 797.}	399.571	0.669081	newly	7800.	14 706.1	{7527., 18 645.}	93.5098	0.282371	Reff	0.972916	1.10254	{0.701504, 1.7828}	31.3517	ar	38 662.	79 224.6	{78 759., 79 585.}	104.916
39	R0	{1.27466, 3.0741}	{1.91324, 6.20811}	{{1.89934, 6.19362}, {1.9862, 6.28417}}	{50.0979, 101.949}	inf	2301.	9627.46	{5535., 13 437.}	318.403	0.602449	newly	4560.	9557.46	{4827., 14 858.}	110.526	0.295229	Reff	0.912975	0.762479	{0.551948, 1.17577}	29.6778	ar	40 181.	77 152.7	{76 332., 79 286.}	92.013
40	R0	{1.33045, 2.68154}	{2.27423, 6.876}	{{2.27423, 6.876}, {2.27423, 6.876}}	{70.9369, 156.42}	inf	3224.	14 612.2	{9854., 18 809.}	355.527	0.646748	newly	6981.	12 975.7	{7751., 19 348.}	88.8531	0.259634	Reff	0.885574	0.77449	{0.472107, 1.34901}	37.976	ar	45 951.	85 776.6	{85 543., 86 036.}	86.6697
41	R0	{1.37003, 2.83984}	{2.33614, 6.89476}	{{2.33614, 6.89476}, {2.33614, 6.89476}}	{70.5174, 142.787}	inf	4061.	17 388.9	{12 618., 20 846.}	330.63	0.623907	newly	8819.	16 356.9	{10 343., 20 718.}	88.5023	0.26852	Reff	0.953239	0.971059	{0.542966, 1.6977}	33.5448	ar	48 926.	86 878.2	{86 567., 87 224.}	77.5707
42	R0	{1.3003, 2.94885}	{2.21225, 8.44061}	{{2.19024, 8.25876}, {2.28593, 9.04943}}	{70.1337, 186.234}	inf	3021.	16 006.5	{11 454., 19 461.}	429.841	0.714999	newly	6589.	12 206.7	{7896., 15 262.}	85.471	0.25671	Reff	0.931561	0.858824	{0.534857, 1.23871}	26.9457	ar	44 237.	84 663.	{83 911., 86 051.}	91.3851
43	R0	{1.33756, 2.92653}	{1.88643, 4.56746}	{{1.88643, 4.56746}, {1.88643, 4.56746}}	{41.0352, 56.0708}	inf	3675.	9485.68	{6395., 13 164.}	158.114	0.395323	newly	8332.	12 303.8	{7053., 19 838.}	55.5162	0.175736	Reff	0.990757	0.782427	{0.57687, 1.18841}	30.3463	ar	47 342.	76 150.	{75 716., 76 617.}	60.8509
44	R0	{1.35801, 2.92276}	{2.40735, 8.12091}	{{2.40735, 8.12091}, {2.40735, 8.12091}}	{77.2704, 177.851}	inf	4090.	18 268.3	{12 246., 21 772.}	352.259	0.657076	newly	8641.	14 703.3	{10 796., 18 383.}	76.9812	0.264513	Reff	0.933387	1.00436	{0.596589, 1.85212}	36.1892	ar	47 940.	87 938.7	{87 707., 88 162.}	83.4349
45	R0	{1.31342, 4.02649}	{1.96104, 8.17067}	{{1.96104, 8.17067}, {1.96104, 8.17067}}	{49.3079, 102.923}	inf	3015.	12 523.9	{9639., 14 535.}	315.385	0.612988	newly	4999.	10 154.2	{7190., 12 262.}	103.125	0.298456	Reff	0.979207	0.903307	{0.63371, 1.29552}	23.7982	ar	42 995.	78 571.4	{78 132., 79 060.}	82.7454
46	R0	{1.37919, 3.75889}	{2.8732, 14.7003}	{{2.86193, 14.6465}, {2.90379, 14.8888}}	{108.325, 291.08}	inf	3990.	23 783.9	{12 748., 28 671.}	497.43	0.749895	newly	7235.	11 656.2	{7950., 13 389.}	67.8768	0.232778	Reff	1.00539	1.34399	{0.722753, 2.31962}	47.5187	ar	49 157.	93 030.8	{92 865., 93 477.}	89.2524
47	R0	{1.37458, 3.56117}	{2.7943, 13.5962}	{{2.7943, 13.5962}, {2.7943, 13.5962}}	{103.284, 281.79}	inf	4168.	23 690.5	{20 543., 27 235.}	470.565	0.731409	newly	7293.	12 077.	{9455., 14 060.}	73.5749	0.250102	Reff	0.937725	1.16471	{0.627879, 1.75074}	42.8914	ar	48 478.	92 425.	{92 272., 92 581.}	90.6535
48	R0	{1.33393, 3.78296}	{2.7811, 19.6712}	{{2.7811, 19.6712}, {2.7811, 19.6712}}	{108.489, 419.995}	inf	3354.	22 653.6	{11 921., 27 265.}	581.096	0.814838	newly	5927.	8310.12	{5268., 9650.}	51.8787	0.216045	Reff	0.973742	1.3964	{0.854521, 2.12985}	49.7446	ar	44 885.	92 343.4	{92 163., 92 489.}	105.733
49	R0	{1.24918, 2.39046}	{1.84836, 6.13642}	{{1.84836, 6.13642}, {1.84836, 6.13642}}	{47.9659, 156.705}	inf	1875.	9896.44	{7113., 12 457.}	427.81	0.706793	newly	4978.	10 114.1	{6473., 14 068.}	104.151	0.293562	Reff	0.958921	0.838948	{0.622417, 1.1133}	25.0856	ar	35 969.	74 960.1	{74 591., 75 406.}	108.402
50	R0	{1.28281, 2.3032}	{1.98388, 6.25659}	{{1.98388, 6.25659}, {1.98388, 6.25659}}	{54.6511, 171.648}	inf	2262.	11 051.2	{7061., 14 237.}	388.559	0.674019	newly	6004.	11 132.5	{7015., 14 744.}	86.5834	0.25651	Reff	0.938273	0.852926	{0.574928, 1.49303}	33.118	ar	40 223.	79 238.6	{78 771., 79 670.}	96.9981
